# Effectiveness of a coordinated ambulatory care program for patients with mental disorders or multiple sclerosis: results of a prospective non-randomized controlled trial in South Germany

**DOI:** 10.3389/fpsyt.2023.1183710

**Published:** 2023-12-19

**Authors:** Tharanya Seeralan, Julia L. Magaard, Alexander Engels, Ramona Meister, Levente Kriston, Sarah Liebherz, Hans-Helmut König, Martin Härter

**Affiliations:** ^1^Department of Medical Psychology, Center for Psychosocial Medicine, University Medical Center Hamburg-Eppendorf, Hamburg, Germany; ^2^Evangelisches Krankenhaus Ginsterhof GmbH, Psychosomatic Clinic, Rosengarten, Germany; ^3^Department of Health Economics and Health Services Research, Center for Psychosocial Medicine, University Medical Center Hamburg-Eppendorf, Hamburg, Germany

**Keywords:** evaluation of effectiveness, mental health care, quality of life, patient-reported outcomes, integrated care, coordinated care, collaborative care, non-randomized controlled trial

## Abstract

**Background:**

The Psychiatry, Neurology, Psychosomatics and Psychotherapy (PNP) program of the German statutory health insurance AOK BW promotes coordinated and evidence-based specialist care with the aim of providing individualized, guideline-based outpatient care, strengthening the collaboration between health care providers, as well as reducing care costs. The purpose of this study was to evaluate its effectiveness regarding patient-reported outcomes compared to the less specialized general practitioner program (GP) and usual care (UC).

**Materials and methods:**

AOK insured patients, who were on sick leave due to a mental disorder (affective disorder, anxiety disorder, adjustment disorder, somatoform disorder, alcohol abuse disorder, schizophrenia) or multiple sclerosis were included in the prospective non-randomized controlled study. All patients either participated in the PNP program (intervention group, IG-PNP), the general practitioner program (control group, CG-GP) or usual care (control group, CG-UC). Entropy balancing was used to adjust for baseline imbalance between groups. Primary outcome was health-related quality of life, assessed by the Short-form health survey (SF-36) 12 months after diagnosis. Secondary outcomes included symptom severity, functional health, and treatment satisfaction.

**Results:**

Of the 14,483 insured patients who were contacted, 1,104 patients participated at baseline and 725 at follow-up. The adjusted mean differences of SF-36 sum score did not significantly differ between groups: −1.89 (95%-CI = −4.60; 0.81, *p* = 0.170) between IG-PNP and CG-GP, and −1.42 (95%-CI = −4.05; 1.22, *p* = 0.293) between PNP and CG-UC. The adjusted mean differences of secondary outcomes did not differ between groups, except for a slightly higher increase of functional health in CG-UC.

**Conclusion:**

We found no evidence that the PNP program is superior to the GP program or to usual care in terms of patient-reported outcomes or treatment satisfaction. The results are limited by the low response rate. Accordingly, future studies should strive for more representative samples. To improve the program, an integration of further collaborative care elements and guideline recommendations might be useful.

**Clinical trial registration:**

DRKS (German Clinical Trials Register https://drks.de/search/en); identifier (DRKS00013114).

## 1 Introduction

Mental and neurological disorders are related to a high degree of personal suffering, disease burden, impaired health, and reduced quality of life (1–3). About 27% of the EU adult population aged 18–65 years is or has been affected by at least one mental disorder in the past 12 months (4). Consequently, mental and neurological disorders represent a major challenge for the health care system (5, 6).

In Germany a well-developed, but fragmented mental health care system exists (7). Large delays between detection and adequate treatment of mental disorders lead to a greater risk to maintain high degrees of burden (8–12). In addition, separated responsibilities of health care providers in different sectors impede cooperation and the transition of patients between sectors (e.g., primary care, specialist care, rehabilitation) (11). Consequently, innovative care networks aim to overcome those intersectoral barriers and to optimize care of patients with mental and neurological disorders. For example, integrating mental health care specialists into primary care helps to substantially improve access and quality of mental health care, especially for those who experience difficulties in engaging in specialized mental health care (13). Clinical practice guidelines recommend stepped and collaborative care models for the treatment of patients with depression (14, 15) and anxiety disorders (16, 17). Collaborative care aims to provide evidence-based treatment by strengthening the cooperation between health care providers. For instance, collaborative care models are superior to less integrated models and usual care among patients with mental disorders such as anxiety disorders (18) and depression (19, 20). The integration of mental health care into primary care can be accomplished in a variety of ways. With a focus on improving coordinated ambulatory care, selective contracts between statutory health insurance companies and health care providers aim to address the problems with fragmented mental health care in accordance with the German Social Security Code V (21). The established “GP program” (“HausarztProgramm”) and the “specialist program” (“FacharztProgramm”) are two of these programs developed and implemented by the German statutory health insurances AOK Baden Wuerttemberg (AOK BW) and Betriebskrankenkasse (Bosch BKK) in Southwestern Germany. The Psychiatry, Neurology, Psychosomatics and Psychotherapy (PNP) contract is part of the “specialist program.” Insurees enrolled in the “specialist program” have the option to receive specialist care within the PNP contract, if they need outpatient care in psychiatry, neurology, psychosomatics and/or psychotherapy. The aim is to provide individualized and guideline-based outpatient care, to strengthen the coordination and collaboration between health care providers, as well as to improve diagnostics. Although the PNP contract was not developed as a collaborative care model, it does follow principles of integrated care by strengthening the collaboration and enhancing the communication between health care providers and care extenders (e.g., social services called “Sozialer Dienst” provided by the AOK BW) (22, 23). Therefore, this selective contract is hereinafter referred to as the “PNP program.” The different components of the PNP program and the GP program compared to usual care are described in the study protocol (23). The PNP program has been comprehensively implemented since 2012 in Baden-Wuerttemberg, South Germany.

The evaluation of structural and process quality of the PNP program showed that the majority of the participating health care providers were satisfied with it (e.g., patient-orientated and treatment of severely ill patients, better access to care, less bureaucracy). Nevertheless, problems with access to care (e.g., delays in enrollment, patients without a primary care physicians participating in the GP program, which was required for enrolment in the specialist program), treatment (e.g., limited capacities of health care providers), and insufficient cooperation were also reported (24). The analyses of health insurance fund data suggested that the PNP program can favorably impact sick pay and sick leave days, but not treatment costs (22). In summary, international evidence shows that integrated care models can lead to long-term improvement in patient-reported outcomes among patients with mental disorders. Hence, the purpose of this study was to evaluate the effectiveness of PNP program regarding patient-reported outcomes compared to the GP program and usual care.

## 2 Materials and methods

### 2.1 Study design

We conducted a prospective non-randomized controlled trial comparing the intervention group, which consisted of patients with access to specialist care within the PNP program (intervention group: IG-PNP) with two control groups. Patients in the first control group participated in the specific GP program, but not the PNP program (CG-GP). Patients in the second control group had access to usual care only (CG-UC). We included consecutively recruited AOK insurees from the three groups between November 2017 and October 2019. We measured health-related quality of life, patient-reported symptom severity and patient satisfaction at baseline (date of sick leave) and at 12-month follow-up. Only participants who gave their informed consent were included. Participants received a compensation of 15€ for returning both questionnaires. The study protocol was published elsewhere (23).

We expected higher health-related quality of life, functional health and patient satisfaction and lower illness-specific symptom burden in the IG-PNP than in the two control groups CG-GP and CG-UC, respectively.

### 2.2 Inclusion and exclusion criteria

We included patients who were on sick leave due to one of the following mental or neurological disorders for the first time during the previous 12 months: affective disorders (F31.x, F32.x, F33.x, F34.1), anxiety disorders (F40.x, F41.x), adjustment disorder (F43.2), somatoform disorders (F45.x), alcohol abuse disorders (F10.x), schizophrenia (F20.x), or multiple sclerosis (G35.x). The timely availability of sick leave diagnoses allowed for recruiting study participants as soon as possible after medical appointments. Patients were eligible, if they were insured by the AOK BW, lived in Baden-Wuerttemberg, were at least 18 years old, and were treated by a health care provider licensed in Baden-Wuerttemberg. We excluded patients who had a legal guardian, lived outside of Baden-Wuerttemberg or died.

### 2.3 Interventions

Table 1 provides an overview of the three health care programs. A more detailed comparative description of the care programs (e.g., differences in organization and payment of health care services) can be found in the study protocol (23).

**Table 1:** Summary of the differences and overlaps between the health care programs.

	Usual care	General practitioners program	PNP program
Patient requirements for participation and gate keeping	– None (patients had potentially free access to health care providers with a license in Baden-Wuerttemberg)	– Patients had to commit (a) to a minimum time of program participation of 1 year and (b) first seek help from a GP enrolled in the GP program (exception: emergencies, gynecologist, ophthalmologists, pediatricians). – Potentially free choice between specialists when referred by the GP	– Patients had to commit to (a) a minimum time of participation of 1 year in the program and (b) first seek help from a GP enrolled in the GP program (exception: emergencies, gynecologist, ophthalmologists, pediatricians). – Access to specialists participating in the specialist program when referred by the GP to avoid mis-indicated specialist consultations and allow for more targeted, coordinated referrals
Role of GPs	– Areas of responsibility: diagnosis, treatment, referral to specialists	– Areas of responsibility: diagnosis, treatment, referral to specialists – GP guided through care: structured coordinating and communication to specialists care and merging results of medical examinations	– Areas of responsibility: diagnosis, treatment, referral to specialists – GP guided through care: structured coordinating and communication to specialists care and merging results of medical examinations
Social service	– Social service of the AOK Baden Wuerttemberg	– More structured cooperation between health care provider and social service of the AOK Baden Wuerttemberg	– More structured cooperation between health care provider and social service of the AOK Baden Wuerttemberg
Quality management	– Mandatory continuous training courses for all providers	– Mandatory continuous training courses for all providers – Participation in quality circles on drug therapy (once per quarter) for GPs	– Mandatory continuous training courses for all providers – Participation in quality circles on drug therapy (once per quarter) for GPs (optional for specialists)
Organization (Psychotherapy)	– Review process for approval of long-term psychotherapy mandatory	– Review process for approval of long-term psychotherapy mandatory	– No review process for approval of long-term psychotherapy needed
Treatment content (Psychotherapy)	– Cognitive Behavioral Therapy – Psychodynamic Psychotherapy – Psychoanalytic therapy – Neuropsychological therapy – Hypnosis – Eye Movement Desensitization and Reprocessing (EMDR)	– Cognitive Behavioral Therapy – Psychodynamic Psychotherapy – Psychoanalytic therapy – Neuropsychological therapy – Hypnosis – Eye Movement Desensitization and Reprocessing (EMDR)	– Cognitive Behavioral Therapy – Psychodynamic Psychotherapy – Psychoanalytic therapy – Additional treatment methods depending on diagnosis: ○ Neuropsychological therapy ○ Hypnosis ○ Eye Movement Desensitization and Reprocessing (EMDR) ○ Systemic psychotherapy ○ Biofeedback ○ Interpersonal therapy
Additional guidelines on accessibility (Psychotherapy)	/	/	– For acute cases: initial session within 3 days; start of psychotherapy within 7 days after established diagnosis – For initial treatment: start of psychotherapy within 4 weeks after established diagnosis
Additional guidelines on accessibility (Psychiatry)	/	/	– Limit of waiting time up to 30 min – For acute cases: first doctor’s appointment within the same day
Additional guidelines on accessibility (Neurology)	/	/	– Limit of waiting time up to 30 min – For acute cases: first doctor’s appointment within the same day

GP, general practitioner program; PNP, specialist program (selective care contract in psychiatry, neurology, psychosomatics and psychotherapy); UC, usual care.

Patients who wish to participate in the PNP program must first be informed about and enrolled in the GP program by their primary care physician. The enrollment in the GP program is mandatory for participation in the PNP program or in other medical specialist’s program offered by the AOK. Both patients in the PNP program and patients in the General practitioner (GP) program had to commit to (a) a minimum time of participation of 1 year in the program and (b) first seek help from a GP enrolled in the GP program (exception: emergencies, gynecologist, ophthalmologists, pediatricians). While patients in the GP program had potentially free choice between specialists when referred by the GP, patients in the PNP program only had access to specialists participating in the specialist program when they were referred by the GP (“gate keeper”). This approach was intended to avoid unnecessary specialist consultations and enable more targeted referrals. The utilization of the intervention, e.g., contact with a PNP specialist, was not a criterion.

Patients in usual care had unrestricted access to health care providers (GP or specialist) with a license in Baden-Wuerttemberg. While this meant that patients in UC had the freedom to see the specialist of their choice, they were also still affected by the less coordinated, less facilitated access conditions as well as intersectoral barriers of routine care (e.g., long wait times for psychotherapy).

### 2.4 Outcome measures

The primary outcome was health-related quality of life (mental component summary score) and was measured by the Short-form health survey (SF-36) (25). Secondary outcomes included functional health (physical component summary score; SF-36) as well as different psychological symptoms measured by the Depression Module (PHQ-9) (26) and Generalized Anxiety Disorder Module (GAD-7) (27) of the Patient Health Questionnaire, by the Somatic Symptom Scale-8 (SSS-8) (28) and by the short-form of Alcohol Use Disorders Identification Test (AUDIT-C) (29). Satisfaction with ambulatory care (ZAPA) (30) was used to measure patient satisfaction with GPs and specialist care at follow-up.

### 2.5 Statistical analysis

A sample size of 536 patients was required to detect a clinically relevant effect regarding the primary outcome between IG-PNP and both control groups (23). We used entropy balancing (31, 32) to control for differences between the groups due to the quasi-experimental design. Entropy balancing is a reweighting method that directly targets balancing the covariate moments between the intervention and control groups. The weights designed to alleviate differences between groups in the mean, variance and skewness in selected patient characteristics were in our study: age, gender, education, residency, employment, period of selection, health-related quality of life, degree of depressive, anxiety and somatoform symptoms, degree of alcohol consumption, days of incapacity to work, mental health services utilization, medication, physical comorbidity, diagnoses of depression, somatoform disorder and anxiety disorder.

We used linear mixed models with fixed effects of group membership, time, their interaction, and further covariates (age, gender, diagnoses of sick leave) to test the effectiveness hypotheses. In a linear mixed model, both different levels that may vary but whose variation is not part of the effect being tested (e.g., person level and practice level) as well as cases that have a valid value in the outcome variable at only one measurement time can be included. Thus, more data can be included in the estimation. To control for potential confounders, the weights from entropy balancing were incorporated by using weighted maximum likelihood in the estimation. We planned to account for clustering of patients within practices, but it turned out to be unnecessary because the average cluster size was much lower than expected (1.2 patients per practice).

## 3 Results

### 3.1 Sample characteristics

**Figure 1:**
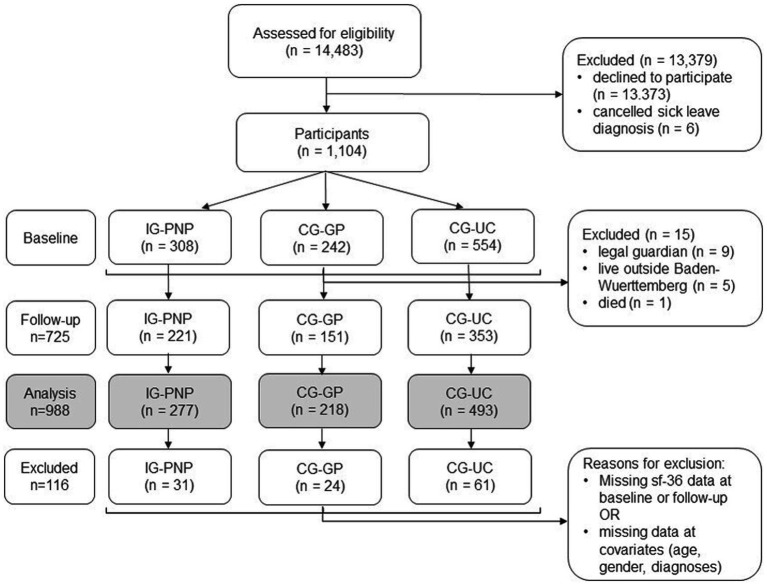
Flow of participants.

Of 14,483 patients contacted, 1,104 responded at baseline (response rate: 8%), and 725 at follow-up (response rate: 5%). Due to model requirements (e.g., complete data on all covariates at baseline), data of 988 participants could be included in the main analysis (Figure 1). From IG-PNP, 1 out of 277 participants (0.4%) switched to CG-GP and 6 (2.2%) to CG-UC; from CG-GP, 19 out of 218 participants (8.7%) switched into IG-PNP and 10 (4.6%) to CG-UC and from CG-UC 3 out of 493 participants (0.6%) switched into IG-PNP and 14 (2.8%) into CG-UC during the study.

On average patients were 46 years old (SD = 12.2) and 61.5% were female. At baseline, 50% were in the CG-UC, 28% in IG-PNP and 22% in CG-GP program. Table 2 shows the differences between the baseline variables before and after entropy balancing. Patients in the IG-PNP were older, less educated, more likely to be on medication due to a mental or neurological disorder, more likely to have more physical comorbidities and had more often depression and anxiety diagnoses. These differences could be minimized by using entropy balancing.

**Table 2:** Unadjusted und adjusted sample characteristics before and after entropy balancing for the primary outcome analysis.

Variables	UC (before EB) *n* = 493	GP (before EB) *n* = 218	PNP *n* = 277	UC (after EB)	GP (after EB)
Age (M, SD)	44.2 (12.77)	45.5 (11.58)	48.6 (11.10)	48.6 (11.10)	48.6 (11.09)
Gender [female] (%)	65.5	66.5	61.7	61.7	61.7
Education^a^ [German Abitur or equivalent] (%)	32.0	27.0	22.7	22.7	22.7
Employment^b^ [full time employment] (%)	63.0	57.7	60.0	60.0	60.0
Residency^c^ [urban] (%)	84.7	77.0	81.7	81.7	81.7
Period of selection [01.04. 2018–30.09.2019] (%)	36.5	35.8	35.0	35.0	35.0
Health-related quality of life at baseline^d^ (M, SD)	29.76 (12.92)	30.18 (13.41)	31.21 (12.29)	31.0 (12.85)	31.62 (11.97)
Degree of depressive symptoms^e^ (M, SD)	12.48 (6.46)	12.20 (6.72)	11.71 (5.82)	12.10 (6.27)	12.28 (6.25)
Degree of anxiety symptoms^f^ (M, SD)	10.29 (5.69)	10.04 (5.72)	10.04 (5.66)	10.18 (5.85)	10.05 (5.57)
Degree of somatoform symptoms^g^ (M, SD)	12.78 (6.51)	12.57 (6.59)	13.44 (6.08)	13.57 (6.42)	13.79 (6.49)
Degree of alcohol consumption^h^ (M, SD)	2.52 (2.33)	2.34 (2.15)	2.36 (2.14)	2.30 (2.15)	2.38 (2.27)
Days of incapacity to work^i^ (M, SD)	43.22 (52.12)	40.26 (42.68)	43.70 (47.05)	44.29 (52.74)	44.34 (48.11)
Mental health services utilization^j^ [yes] (%)	60.6	59.0	63.7	63.7	63.7
Hospital stay due to mental or neurological illness^k^ [yes] (%)	13.2	12.5	14.1	14.3	13.8
Medication due to mental or neurological illness^l^ [yes] (%)	46.8	45.2	55.1	55.7	55.3
Physical comorbidity (number of diagnoses) (M, SD)	1.39 (1.68)	1.29 (1.52)	2.0 (1.98)	2.0 (1.99)	2.0 (1.98)
Diagnosis of depression [yes] (%)	79.5	79.8	88.4	88.4	88.4
Diagnosis of somatoform disorder [yes] (%)	46.7	34.9	43.7	43.7	43.7
Diagnosis of anxiety disorder [yes] (%)	30.6	23.4	32.1	32.1	32.1

GP, general practitioner program; PNP, specialist program (selective care contract in psychiatry, neurology, psychosomatics and psychotherapy); UC, usual care; EB, entropy balancing; M, mean value; SD, standard deviation. ^a^*n* = 956, ^b^*n* = 974, ^c^*n* = 958, ^d^*n* = 928, ^e^*n* = 977, ^f^*n* = 978, ^g^*n* = 976, ^h^*n* = 957, ^i^*n* = 938, ^j^*n* = 977, ^k^*n* = 965, ^l^*n* = 965.

### 3.2 Dropout

In comparison to all patients contacted at baseline, study participants were more likely to be older, female, to have more than one mental co-diagnosis and less likely to have another citizenship than the German one (Supplementary Additional File 1: Supplementary Table 1). Eligible patients and participants at baseline had similar average days of incapacity to work in the past 12 months. There were no relevant differences regarding demographic variables, health status and health services use at baseline between patients with and without follow-up data (Supplementary Additional File 1: Supplementary Table 2).

### 3.3 Effectiveness

There was no significant change in health-related quality of life at follow-up between groups. The model showed only negligible differences with regard to the average change over time. The decline over time was slightly, but not significantly, stronger in IG-PNP when compared to CG-GP [−1.89; 95%-confidence interval (CI) = −4.60; 0.81, *p* = 0.170) or CG-UC (−1.42; 95%-CI = −4.05; 1.22, *p* = 0.293] (Table 3). Regarding secondary outcomes, the PNP program did not yield significant improvements in functional health, depressive symptoms, anxiety symptoms, somatoform symptoms and alcohol consumption during the 12-month follow-up period. However, we found that insurees in CG-UC care achieved slightly higher functional health over time than insurees in the IG-PNP (MD 2.24, 95%-CI = −0.33; 4.16, *p* = 0.022) (Table 3). Since the average difference over time with regard to the outcomes was of main interest, we only display interaction terms in Table 3 as well as estimated marginal means (EMM) between baseline (t0) and 12-month follow-up (t1) in Table 4. Additionally, main effects of all outcomes can be found in Supplementary Additional File 3: Supplementary Tables 1–6.

**Table 3:** Estimated mean differences based on the mixed linear model.

Outcome	Parameter	Estimate	Ste	DF	*t*-value	*p*-value	95%-CI	
							Lower	Upper
Health-related quality of life	Time * UC (vs. PNP) (interaction term)	−1.42	1.34	987	−1.05	0.293	−4.05	1.22
	Time * GP (vs. PNP) (interaction term)	−1.89	1.38	987	−1.37	0.170	−4.60	0.81
Functional health	Time * UC (vs. PNP) (interaction term)	2.24	0.98	987	2.30	0.022*	0.33	4.16
	Time * GP (vs. PNP) (interaction term)	−0.23	1.00	987	−0.22	0.822	−2.19	1.74
Depressive symptoms	Time * UC (vs. PNP) (interaction term)	0.05	0.58	1,026	0.08	0.937	−1.09	1.18
	Time * GP (vs. PNP) (interaction term)	0.37	0.59	1,026	0.63	0.530	−0.79	1.53
Anxiety symptoms	Time * UC (vs. PNP) (interaction term)	0.69	0.52	1,025	1.33	0.185	−0.33	1.70
	Time * GP (vs. PNP) (interaction term)	0.96	0.53	1,025	1.82	0.069	−0.07	2.00
Somatoform symptoms	Time * UC (vs. PNP) (interaction term)	0.00	0.54	1,026	−0.01	0.994	−1.06	1.05
	Time * GP (vs. PNP) (interaction term)	0.06	0.55	1,026	0.10	0.918	−1.02	1.13
Alcohol consumption	Time * UC (vs. PNP) (interaction term)	−0.07	0.15	1,016	−0.48	0.629	−0.38	0.23
	Time * GP (vs. PNP) (interaction term)	0.04	0.16	1,016	0.25	0.800	−0.27	0.35

GP, general practitioner program; PNP, specialist program (selective care contract in psychiatry, neurology, psychosomatics and psychotherapy, reference group PNP coded with 0); UC, usual care; Ste standard error; DF, degrees of freedom; CI, confidence interval. * and bold = significant with a *p*-value ≤ 0.05.

**Table 4:** Estimated marginal means between baseline (t_0_) and 12-month follow-up (t_1_) based on the mixed linear model.

Outcome	Group	t0	t1
		EMM	EMM
Health-related quality of life	GP	31.44	37.50
	UC	31.05	37.59
	PNP	31.26	39.21
Functional health	GP	44.46	44.10
	UC	43.65	45.76
	PNP	44.03	43.90
Depressive symptoms	GP	11.94	10.20
	UC	12.12	10.06
	PNP	11.88	9.77
Anxiety symptoms	GP	9.82	8.07
	UC	10.16	8.13
	PNP	10.09	7.38
Somatoform symptoms	GP	12.84	11.82
	UC	13.07	11.99
	PNP	13.41	12.33
Alcohol consumption	GP	2.37	2.19
	UC	2.46	2.17
	PNP	2.41	2.20

GP, general practitioner program; PNP, specialist program (selective care contract in psychiatry, neurology, psychosomatics and psychotherapy, reference group PNP coded with 0); UC, usual care; EMM, estimated marginal mean.

### 3.4 Patient satisfaction with outpatient care

There were no differences regarding the adjusted means of patient satisfaction with general practitioners’ care (Supplementary Additional File 3: Supplementary Table 7), with specialized outpatient care (Supplementary Additional File 3: Supplementary Table 8) or with outpatient psychotherapy (Supplementary Additional File 3: Supplementary Table 9) at follow-up between the groups.

### 3.5 Additional analyses

To find out if specific subgroups benefit from the PNP program additional moderator analyses were performed by adding interaction terms (moderator variable*time*group) to the model. The explorative results indicate that insurees on sick leave due to multiple sclerosis may benefit more from the PNP program than UC (MD −14.99, 95%-CI = −29.71; −0.27, *p* = 0.046) regarding the primary outcome. Insurees with lower health-related quality of life at baseline (MD 0.19, 95%-CI = 0.03; 0.35, *p* = 0.020) and lower physical comorbidity (MD 1.80, 95%-CI = 0.28; 3.32, *p* = 0.021) may benefit more from the PNP program-contract than from GP-centered care (Supplementary Additional File 2).

## 4 Discussion

This prospective non-randomized controlled study examined the effectiveness of the participation in the new PNP program regarding health-related quality of life and patient satisfaction compared to a specific GP program and usual care among patients with mental disorder and multiple sclerosis on sick-leave. We found no significant differences between the IG-PNP and the control groups regarding the change of health-related quality of life, symptom burden (depressive, anxiety, somatoform symptoms, or alcohol consumption) after 1 year of inclusion. We also found no significant differences regarding patient satisfaction with received care at follow-up. However, there was a slightly higher significant increase of functional health over time in CG-UC than in IG-PNP, which does not align with the hypothesis of this study. It also contrasts with previous national and international findings on the superiority of coordinated and collaborative care-based interventions among patients with mental disorders (18–20, 33–36).

Findings based on health insurance fund data show that the PNP program favorably influenced sick leave days (22). Accordingly, we assumed that health-related quality of life or functional health are closely related to work ability, but we found no improvement in these patient-reported measures.

One possible explanation is that the study samples may be different. Due to drop-out of male patients, younger patients, patients with another citizenship than the German one and with less mental comorbidities, these patients were underrepresented in the current study, whereas there is no observed dropout among the study based on health insurance fund data (22). Furthermore, it is possible, that the specialist program favorably influences sick leave days, but the impact on health-related quality of life and functional health assessed by patient-reported outcomes was so far not measurable. One further reason might result from problems with the use of guideline-based care: Although the intervention group potentially had free access to different health services related to the PNP contract, this did not ensure that all patients of the intervention group actually received or used PNP treatment, nor did it verify the extent and appropriateness (e.g., guideline-based recommendations) of the treatments provided in the PNP intervention group. Results based on health insurance fund data indicate, that a relevant proportion of patients with depression in the GP and PNP program were still untreated or insufficiently treated during the first 12 months after sick leave (37). Although waiting times for acute and severely ill cases were lower in the PNP program compared to the control groups, it is not clear whether access to care is actually facilitated for participants of the PNP program. Reasons for this included limited capacities of treatment providers, delays in enrolment in the specialist program, and the fact that care providers did not always meet the requirements (e.g., GP without participation in the GP program) (24).

For practitioners, intensified training or education as a preparation as well as regular network meetings may be useful to strengthen and improve cooperation, and also contribute to quality improvement and assurance (38). Further, cooperation and communication among practitioners can also be enhanced through the support of case managers (39), liaison with community psychiatric teams (40) or through enabling digital communication approaches (e.g., sharing electronic patient records to foster the exchange of information). In order to reach chronically ill patients who remain on sick leave for a longer period of time or suffer from recurrent mental illness, systematic screening and early enrolment in the specialist program could be considered. Improving psychoeducation and health competence of patients can be helpful in this respect. Further, the use of systematic monitoring of the course of treatment and the systematic recording of treatment results can possibly increase the effectiveness of the PNP program. Based on the existing literature on integrated and collaborative care models, the PNP program is most likely to be placed at the “lower level” of collaboration (41, 42). In order to strengthen the collaboration and its effectiveness compared to other care models, the PNP program could benefit from (a) an increased integration and expansion of collaborative care elements in order to favorably impact patient benefit and practitioner commitment and (b) a diligent implementation and application of a more comprehensive collaborative care approach (18).

### 4.1 Strengths and limitations

The prospective non-randomized controlled trial was designed to evaluate the target intervention within the context of the complex health care reality. Although randomized controlled trials could provide the best evidence in evaluation studies (43), due to ethical and contractual matters randomization was not possible. All insurees of the AOK BW already decided voluntarily to participate in the GP program and in the specialized PNP program. Therefore, we cannot exclude possible selection biases. In order to minimize the risk of bias, we controlled for demographic and clinical variables using entropy balancing as well as adjustment through covariates. An intention-to-treat approach was realized to evaluate if having access to care based on the PNP program has a positive effect on (mental) health of patients compared to having access to the GP program only or to usual care. Using this approach, our results are limited to the comparison of the different health programs and we cannot derive evidence for the effectiveness of the different treatments within the programs.

The evaluation of different patient-reported outcomes is a major strength of this study compared to health insurance fund data or data from the health care providers. The high dropout rates limit generalizability of the study results. Male patients, younger patients, patients with another citizenship than the German one and with less mental comorbidities were underrepresented in our study. Possible reasons are language barriers, aspects of gender socialization and less motivation because of less identification as mentally ill person. Furthermore, it should be mentioned that the results of the moderator analyses are limited due to alpha-inflation, because alpha was not adjusted for the number of potential outcomes. Thus, there is an increased risk of false positive results, which is in particular true for the various subgroup analysis that pertain to very small samples (e.g., patients with multiple sclerosis). Therefore, we point out that they should be considered as exploratory results only. For robust results, further research should consider equally sized subgroups.

In addition, the results are limited to patients with a new sick leave due to a mental disorder or multiple sclerosis. Accordingly, we cannot generalize our results to patients without sick leaves, patients with recurred or chronic sick leaves as well as to retired patients or children.

## 5 Conclusion

We found no clear evidence that the PNP program is superior to the GP program or to usual care in terms of effectiveness. Patients in the PNP program reported similar levels of health-related quality of life 1 year after sick leave due to a mental disorder or multiple sclerosis in comparison to patients in the GP program or in usual care. Initial health-related quality of life, physical comorbidity and sick leave due to a diagnosis of multiple sclerosis may have an impact on the relation between change of quality of live within 12-months and group. In addition, there were no differences regarding functional health, symptom burden and satisfaction with outpatient care at follow-up between the PNP program, the GP program and usual care. Despite defined inclusion criteria and entropy balancing, the results are limited by the low response rate and the possibility of an insufficiently controlled selection bias due to relevant unobserved confounders.

## Data availability statement

The datasets presented in this article are not readily available because of missing permission from participants to share anonymized participant data publicly. Requests to access the datasets should be directed to the corresponding author.

## Ethics statement

The study involving humans was approved by the Ethics Committee of the Medical Chamber of Hamburg on 22 September 2017 (reference number: PV5621). We confirm that the study and all methods were carried out in accordance with relevant guidelines and regulations of the Declaration of Helsinki and in accordance with the local legislation and institutional requirements. The participants provided their written informed consent to participate in this study.

## Author contributions

MH, LK, JM, RM, and TS had full access to all data in the study and take responsibility for the integrity of the data and the accuracy of the data analysis. AE, MH, H-HK, LK, SL, JM, RM, and TS contributed to the study concept and design and interpretation of data. MH, JM, and TS contributed to acquisition of data. AE, MH, LK, JM, RM, and TS contributed to analysis of data. TS and JM drafted the manuscript. MH, JM, and H-HK contributed to the study supervision. All authors contributed to critical revision of the manuscript for important intellectual content and approved the final article.

## Supplementary material

SUPPLEMENTARY ADDITIONAL FILE 1 Dropout analyses.

SUPPLEMENTARY ADDITIONAL FILE 2 Moderator analyses.

SUPPLEMENTARY ADDITIONAL FILE 3 Estimated mean differences based on the mixed linear model for theprimary and secondary outcomes.
