# Supplementary material for: Effectiveness of a coordinated ambulatory care program for patients with mental disorders or multiple sclerosis: results of a prospective non-randomized controlled trial in South Germany
Source: Front Psychiatry. 2023 Dec 19;14:1183710. doi: 10.3389/fpsyt.2023.1183710 (PMC10766382; doi:10.3389/fpsyt.2023.1183710)
Supplement: SUPPLEMENTARY ADDITIONAL FILE 1 — Dropout analyses. [file Table_1.DOCX]

Supplementary Material

Additional file 1: Dropout analyses

**Table 1: Dropout eligible patients vs. participants at baseline.**

| variables | group | participants at baseline (n=1.110) | eligible patients (n=13.373) |
| --- | --- | --- | --- |
| age (M, SD) | GP UC PNP | 45.3 (11.7) 44.4 (12.6) 48.8 (10.9) | 41.5 (12.9)  40.2 (12.9)  46.1 (11.9) |
| gender [female] (%) | GP  UC PNP | 65.8 66.2 62.6 | 55.7 55.4 58.4 |
| citizenship [other than German] (%) | GP UC PNP | 8.9 13.6 10.8 | 25.9 27.8 24.9 |
| days of incapacity to work in the past 12 months (M, SD) | GP UC PNP | 23.5 (27.3) 20.9 (30.4) 28.0 (37.3) | 22.1 (27.6) 18.7 (26.2) 27.7 (34.8) |
| number of comorbid mental diagnoses in the past 12 months [one] (%) | GP UC PNP | 26.9 24.1 19.3 | 26.8 26.3 24.2 |
| number of comorbid mental diagnoses in the past 12 months [more than one] (%) | GP UC PNP | 48.5 56.7 64.7 | 40.2 41.8 53.4 |

GP general practitioner program, PNP specialist program (selective care contract in the fields of psychiatry, neurology, psychosomatics and psychotherapy), UC usual care, M mean value, SD standard deviation.

Table 2: Sample characteristics at baseline (t0) and 12-month follow-up (t1).

| Variables | group | n (t1) | participants (t1) (n=725) | n (t0) | participants (t0) (n=1.104) |
| --- | --- | --- | --- | --- | --- |
| age (M, SD) | GP UC PNP | 148 343 217 | 47.0 (11.1) 45.4 (12.4) 49.1 (10.9) | 228 520 292 | 45.4 (11.6) 44.6 (12.7) 48.8 (11.1) |
| gender [female] (%) | GP UC PNP | 148 345 218 | 68.9 68.1 62.4 | 230 528 295 | 66.1 65.5 61.4 |
| days of incapacity to work in the past 12 months (M, SD) | GP UC PNP | 139 333 213 | 32.6 (25.7) 44.6 (63.8) 41.4 (44.2) | 222 503 284 | 39.0 (41.6) 42.7 (52.8) 45.7 (51.4) |
| medication due to mental or neurological illness [yes] (%) | GP UC PNP | 145 345 217 | 42.8 48.4 58.1 | 225 525 292 | 46.7 46.1 55.5 |
| hospital stay due to mental or neurological illness [yes] (%) | GP UC PNP | 149 343 215 | 10.7 14.0 13.5 | 229 520 288 | 13.1 13.5 14.9 |
| mental health services utilization [yes] (%) | GP UC PNP | 150 351 217 | 58.0 64.1 66.8 | 232 532 291 | 59.1 60.3 63.6 |
| health-related quality of life (M, SD) | GP UC PNP | 133 326 195 | 31.0 (13.8) 29.2 (12.8) 31.2 (11.9) | 208 489 260 | 30.3 (13.4) 29.9 (13.0) 31.4 (12.2) |
| functional health  (M, SD) | GP UC PNP | 133 326 195 | 46.2 (10.9) 45.3 (11.3) 43.5 (11.2) | 208 489 260 | 45.1 (11.3) 44.9 (11.1) 43.4 (10.8) |
| degree of depressive symptoms (M, SD) | GP UC PNP | 150 350 217 | 11.3 (6.4) 12.5 (6.4) 11.6 (5.8) | 223 532 291 | 12.2 (6.7) 12.5 (6.4) 11.8 (6.0) |
| degree of anxiety symptoms (M, SD) | GP UC PNP | 150 350 218 | 9.4 (5.5) 10.4 (5.7) 9.9 (5.6) | 233 531 293 | 10.0 (5.7) 10.4 (5.7) 10.1 (5.7) |
| degree of somatoform symptoms (M, SD) | GP UC PNP | 149 349 218 | 11.9 (6.7) 12.8 (6.6) 13.3 (6.0) | 231 530 293 | 12.6 (6.6) 12.9 (6.6) 13.5 (6.2) |
| hazardous alcohol consumption [yes]^1^ (%) | GP UC PNP | 144 346 214 | 10.4 18.8 14.5 | 224 523 286 | 12.9 18.9 15.7 |
| sick leave due to depression (%) | GP UC PNP | 151 353 221 | 65.6 58.6 62.9 | 242 554 308 | 66.1 59.7 63.3 |
| sick leave due to somatoform disorder (%) | GP UC PNP | 151 353 221 | 21.2 28.9 22.6 | 242 554 308 | 19.8 28.0 22.4 |
| sick leave due to anxiety disorder (%) | GP UC PNP | 151 353 221 | 10.6 16.1 15.8 | 242 554 308 | 11.2 14.3 17.5 |
| sick leave due to alcohol abuse disorder (%) | GP UC PNP | 151 353 221 | 2.6 1.1 0.9 | 242 554 308 | 2.5 1.3 0.6 |
| sick leave due to multiple sclerosis (%) | GP UC PNP | 151 353 221 | 4.0 2.8 2.7 | 242 554 308 | 3.7 3.2 2.6 |
| sick leave due to schizophrenia (%) | GP UC PNP | 151 353 221 | 0.0 2.0 2.3 | 242 554 308 | 0.0 1.4 1.9 |
| sick leave due to bipolar disorder (%) | GP UC PNP | 151 353 221 | 0.0 0.0 0.5 | 242 554 308 | 0.2 0.4 0.5 |

GP general practitioner program, PNP specialist program (selective care contract in psychiatry, neurology, psychosomatics and psychotherapy), UC usual care, M mean value, SD standard deviation, ^1^ Note: According to AUDIT-C hazardous alcohol consumption is defined as a score of ≥ 4 for women and for men of ≥ 5.
