# Supplementary material for: Effectiveness of a coordinated ambulatory care program for patients with mental disorders or multiple sclerosis: results of a prospective non-randomized controlled trial in South Germany
Source: Front Psychiatry. 2023 Dec 19;14:1183710. doi: 10.3389/fpsyt.2023.1183710 (PMC10766382; doi:10.3389/fpsyt.2023.1183710)
Supplement: SUPPLEMENTARY ADDITIONAL FILE 2 — Moderator analyses. [file Table_2.DOCX]

Supplementary Material

Additional file 2: Moderator analyses

**Table 1: Results of linear mixed models with the primary outcome and interaction effects between time, group and moderators.**

|  | interaction terms | Estimate | Ste | DF | t-value | p-value | 95%-  confidence interval | |
| --- | --- | --- | --- | --- | --- | --- | --- | --- |
| Illness severity and chronicity | time* UC * sick leave due to alcohol abuse disorder | -11.46 | 12.32 | 987 | -0.93 | 0.352 | -35.63 | 12.71 |
|  | time * GP * sick leave due to alcohol abuse disorder | 8.43 | 11.32 | 987 | 0.74 | 0.457 | -13.78 | 30.64 |
|  | time* UC * sick leave due to schizophrenia | -1.98 | 11.16 | 987 | -0.18 | 0.859 | -23.88 | 19.91 |
|  | time * GP * sick leave due to schizophrenia | ^a^ | ^a^ | ^a^ | ^a^ | ^a^ | ^a^ | ^a^ |
|  | time * UC * sick leave due to depression | 4.78 | 2.76 | 987 | 1.73 | 0.084 | -0.65 | 10.20 |
|  | time * GP * sick leave due to depression | -0.56 | 2.81 | 987 | -0.20 | 0.843 | -6.08 | 4.96 |
|  | time * UC * sick leave due to anxiety disorder | 0.71 | 3.61 | 987 | 0.20 | 0.844 | -6.37 | 7.79 |
|  | time * GP * sick leave due to anxiety disorder | 6.17 | 3.87 | 987 | 1.59 | 0.111 | -1.42 | 13.77 |
|  | time * UC * sick leave due to somatoform disorder | 0.50 | 3.15 | 987 | 0.16 | 0.873 | -5.69 | 6.70 |
|  | time * GP * sick leave due to somatoform disorder | -1.15 | 3.27 | 987 | -0.35 | 0.726 | -7.56 | 5.27 |
|  | time * UC * sick leave due to multiple sclerosis | -14.99 | 7.50 | 987 | -2.00 | **.046*** | -29.71 | -0.27 |
|  | time * GP * sick leave due to multiple sclerosis | -10.56 | 7.53 | 987 | -1.40 | .161 | -25.35 | 4.22 |
|  | time * UC * degree of depressive symptoms | 0.25 | 0.20 | 976 | 1.29 | 0.198 | -0.13 | 0.64 |
|  | time * GP * degree of depressive symptoms | -0.06 | 0.20 | 976 | -0.31 | 0.758 | -0.46 | 0.34 |
|  | time * UC * degree of anxiety symptoms | 0.03 | 0.21 | 977 | 0.14 | 0.889 | -0.38 | 0.44 |
|  | time * GP * degree of anxiety symptoms | -0.37 | 0.22 | 977 | -1.64 | 0.102 | -0.81 | 0.07 |
|  | time * UC * degree of alcohol consumption | -0.34 | 0.62 | 956 | -0.54 | 0.590 | -1.56 | 0.89 |
|  | time * GP * degree of alcohol consumption | 0.08 | 0.65 | 956 | 0.13 | 0.900 | -1.19 | 1.35 |
|  | time * UC * degree of somatoform symptoms | 0.08 | 0.21 | 975 | 0.39 | 0.698 | -0.33 | 0.49 |
|  | time * GP * degree of somatoform symptoms | -0.01 | 0.22 | 975 | -0.04 | 0.970 | -0.43 | 0.42 |
|  | time* UC * days of incapacity to work | 0.01 | 0.03 | 937 | 0.42 | 0.672 | -0.04 | 0.06 |
|  | time* GP * days of incapacity to work | -0.01 | 0.04 | 937 | -0.19 | 0.853 | -0.08 | 0.07 |
|  | time* UC * physical comorbidity | 0.21 | 0.66 | 987 | 0.32 | 0.750 | -1.08 | 1.50 |
|  | time* GP * physical comorbidity | 1.80 | 0.78 | 987 | 2.32 | **0.021*** | 0.28 | 3.32 |
|  | time* UC * health-related quality of life at baseline | 0.05 | 0.08 | 927 | 0.59 | 0.558 | 0.05 | -0.11 |
|  | time* GP * health-related quality of life at baseline | 0.19 | 0.08 | 927 | 2.33 | **0.020*** | 0.03 | 0.35 |
|  | time* UC * illness duration | 0.01 | 0.01 | 814 | 0.92 | 0.360 | -0.01 | 0.02 |
|  | time* GP * illness duration | -0.01 | 0.01 | 814 | -1.46 | 0.143 | -0.03 | 0.00 |
| demo-graphic variables | time* UC *age | -0.14 | 0.12 | 987 | -1.12 | .263 | -0.37 | 0.10 |
|  | time* GP *age | -0.08 | 0.13 | 987 | -0.63 | .530 | -0.33 | 0.17 |
|  | time* UC *gender | -3.68 | 2.77 | 987 | -1.33 | 0.183 | -9.11 | 1.74 |
|  | time* GP *gender | -0.79 | 2.85 | 987 | -0.28 | 0.781 | -6.39 | 4.80 |
|  | time* UC *education | 2.28 | 3.10 | 955 | 0.73 | 0.463 | -3.81 | 8.37 |
|  | time* GP *education | -3.75 | 3.27 | 955 | -1.15 | 0.252 | -10.16 | 2.66 |

GP general practitioner program, PNP specialist program (selective care contract in psychiatry, neurology, psychosomatics and psychotherapy), UC usual care, Ste standard error, DF degrees of freedom, *****p <.05 ^a^ The parameters of the interaction term could not be estimated due to lack of variance.

**Notes:* We used mixed linear models with fixed effects of group membership (IG-PNP / CG-GP / CG-UC) and further covariates (age, gender, mental or neurological diagnoses of sick leave) and added the displayed 36 interaction terms. The parameters of the interaction terms with sick leave due to adjustment disorder and sick leave due to bipolar disorder could not be estimated due to lack of variance.
