# Supplementary material for: Effectiveness of a coordinated ambulatory care program for patients with mental disorders or multiple sclerosis: results of a prospective non-randomized controlled trial in South Germany
Source: Front Psychiatry. 2023 Dec 19;14:1183710. doi: 10.3389/fpsyt.2023.1183710 (PMC10766382; doi:10.3389/fpsyt.2023.1183710)
Supplement: SUPPLEMENTARY ADDITIONAL FILE 3 — Estimated mean differences based on the mixed linear model for theprimary and secondary outcomes. [file Table_3.DOCX]

Supplementary Material

Additional file 3: Estimated mean differences based on the mixed linear model for the primary and secondary outcomes

**Table 1: Estimated mean differences based on the mixed linear model for health-related quality of life.**

|  | Estimate | Ste | DF | t-value | p-value | 95%-CI |
| --- | --- | --- | --- | --- | --- | --- |
| intercept | 33.26 | 2.29 | 987 | 14.51 | <.001 | 28.76; 37.76 |
| UC vs. PNP | -0.20 | 1.04 | 987 | -0.20 | .845 | -2.24; 1.83 |
| GP vs. PNP | 0.18 | 1.13 | 987 | 0.16 | .875 | -2.04; 2.40 |
| time [t1] | 7.95 | 0.94 | 987 | 8.44 | <.001 | 6.10; 9.80 |
| age | 0.04 | 0.03 | 987 | 1.16 | .247 | -0.03; 0.10 |
| gender [male] | 0.18 | 0.79 | 987 | 0.22 | .823 | -1.38; 1.74 |
| sick leave due to depression | -5.80 | 1.44 | 987 | -4.02 | <.001 | -8.63; -2.97 |
| sick leave due to somatoform disorder | 0.19 | 1.50 | 987 | 0.13 | .090 | -2.75; 3.13 |
| sick leave due to anxiety disorder | -3.10 | 1.44 | 987 | -2.15 | .032 | -5.93; -0.27 |
| sick leave due to alcohol abuse disorder | 1.47 | 3.35 | 987 | 0.44 | .660 | -5.10; 8.04 |
| sick leave due to multiple sclerosis | 4.25 | 2.44 | 987 | 1.74 | .082 | -0.54; 9.04 |
| sick leave due to schizophrenia | 1.44 | 3.52 | 987 | 0.41 | .682 | -5.46; 8.35 |
| sick leave due to bipolar disorder | -3.26 | 4.93 | 987 | -0.66 | .508 | -12.93; 6.41 |
| time * UC (vs. PNP) (interaction term) | -1.42 | 1.34 | 987 | -1.05 | .293 | -4.05; 1.22 |
| time * GP (vs. PNP) (interaction term) | -1.89 | 1.38 | 987 | -1.37 | .170 | -4.60; 0.81 |

GP general practitioner program, PNP specialist program (selective care contract in psychiatry, neurology, psychosomatics and psychotherapy, reference group PNP coded with 0), UC usual care, Ste standard error, DF degrees of freedom, CI confidence interval.

**Table 2: Estimated mean differences based on the mixed linear model for the secondary outcome: functional health.**

|  | Estimate | Ste | DF | t-value | p-value | 95%-CI | |
| --- | --- | --- | --- | --- | --- | --- | --- |
| intercept | 57.78 | 1.95 | 987 | 29.59 | <.001 | 53.95; | 61.62 |
| UC vs. PNP | -0.38 | 0.85 | 987 | -0.45 | .656 | -2.05; | 1.29 |
| GP vs. PNP | 0.43 | 0.96 | 987 | 0.45 | .654 | -1.45; | 2.31 |
| time [t1] | -0.13 | 0.68 | 987 | -0.19 | .852 | -1.47; | 1.21 |
| age | -0.29 | 0.03 | 987 | -9.99 | <.001 | -0.34; | -0.23 |
| gender [male] | 0.33 | 0.69 | 987 | 0.49 | .626 | -1.01; | 1.68 |
| sick leave due to alcohol abuse disorder | 1.89 | 2.95 | 987 | 0.64 | .521 | -3.89; | 7.68 |
| sick leave due to schizophrenia | 2.33 | 2.99 | 987 | 0.78 | .436 | -3.54; | 8.20 |
| sick leave due to bipolar disorder | -0.63 | 4.19 | 987 | -0.15 | .880 | -8.86; | 7.59 |
| sick leave due to depression | -0.03 | 1.24 | 987 | -0.03 | .978 | -2.47; | 2.40 |
| sick leave due to anxiety disorder | 0.04 | 1.24 | 987 | 0.03 | .973 | -2.39; | 2.47 |
| sick leave due to somatoform disorder | -2.62 | 1.29 | 987 | -2.04 | .042 | -5.15; | -0.10 |
| sick leave due to multiple sclerosis | -4.50 | 2.11 | 987 | -2.14 | .033 | -8.63; | -0.36 |
| time * UC (vs. PNP) (interaction term) | 2.24 | 0.98 | 987 | 2.30 | **.022*** | 0.33; | 4.16 |
| time * GP (vs. PNP) (interaction term) | -0.23 | 1.00 | 987 | -0.22 | .822 | -2.19; | 1.74 |

GP general practitioner program, PNP specialist program (selective care contract in psychiatry, neurology, psychosomatics and psychotherapy, reference group PNP coded with 0), UC usual care, Ste standard error, DF degrees of freedom, CI confidence interval.

**Table 3: Estimated mean differences based on the mixed linear model for the secondary outcome: depressive symptoms.**

|  | Estimate | Ste | DF | t-value | p-value | 95%-CI | |
| --- | --- | --- | --- | --- | --- | --- | --- |
| intercept | 11.07 | 1.12 | 1026 | 9.90 | <.001 | 8.87; | 13.26 |
| UC vs. PNP | 0.25 | 0.48 | 1026 | 0.51 | .609 | -0.70; | 1.19 |
| GP vs. PNP | 0.06 | 0.54 | 1026 | 0.12 | .904 | -0.99; | 1.12 |
| time [t1] | -2.11 | 0.41 | 1026 | -5.21 | <.001 | -2.91; | -1.32 |
| age | -0.01 | 0.02 | 1026 | -0.85 | .393 | -0.05; | 0.02 |
| gender [male] | -0.35 | 0.39 | 1026 | -0.90 | .371 | -1.11; | 0.41 |
| sick leave due to alcohol abuse disorder | -1.53 | 1.66 | 1026 | -0.92 | .357 | -4.80; | 1.73 |
| sick leave due to schizophrenia | -0.09 | 1.69 | 1026 | -0.05 | .959 | -3.40; | 3.23 |
| sick leave due to bipolar disorder | 1.49 | 2.47 | 1026 | 0.60 | .547 | -3.36; | 6.34 |
| sick leave due to depression | 2.37 | 0.71 | 1026 | 3.34 | .001 | 0.98; | 3.76 |
| sick leave due to anxiety disorder | 0.69 | 0.71 | 1026 | 0.97 | .331 | -0.71; | 2.09 |
| sick leave due to somatoform disorder | 0.11 | 0.74 | 1026 | 0.15 | .878 | -1.33; | 1.56 |
| sick leave due to multiple sclerosis | -0.54 | 1.21 | 1026 | -0.45 | .653 | -2.93; | 1.84 |
| time * UC (vs. PNP) (interaction term) | 0.05 | 0.58 | 1026 | 0.08 | .937 | -1.09; | 1.18 |
| time * GP (vs. PNP) (interaction term) | 0.37 | 0.59 | 1026 | 0.63 | .530 | -0.79 | 1.53 |

GP general practitioner program, PNP specialist program (selective care contract in psychiatry, neurology, psychosomatics and psychotherapy, reference group PNP coded with 0), UC usual care, Ste standard error, DF degrees of freedom, CI confidence interval.

**Table 4: Estimated mean differences based on the mixed linear model for the secondary outcome: anxiety symptoms.**

|  | Estimate | Ste | DF | t-value | p-value | 95%-CI | |
| --- | --- | --- | --- | --- | --- | --- | --- |
| intercept | 8.92 | 1.01 | 1025 | 8.85 | <.001 | 6.94; | 10.89 |
| UC vs. PNP | 0.07 | 0.43 | 1025 | 0.15 | .879 | -0.78; | 0.92 |
| GP vs. PNP | -0.27 | 0.48 | 1025 | -0.55 | .581 | -1.22; | 0.68 |
| time [t1] | -2.71 | 0.36 | 1025 | -7.49 | <.001 | -3.42; | -2.00 |
| age | -0.01 | 0.01 | 1025 | -0.38 | .707 | -0.03; | 0.02 |
| gender [male] | -0.07 | 0.35 | 1025 | -0.21 | .831 | -0.76; | 0.61 |
| sick leave due to alcohol abuse disorder | -1.49 | 1.51 | 1025 | -0.99 | .324 | -4.44; | 1.47 |
| sick leave due to schizophrenia | 1.05 | 1.47 | 1025 | 0.72 | .474 | -1.83; | 3.93 |
| sick leave due to bipolar disorder | 1.59 | 2.20 | 1025 | 0.72 | .470 | -2.72; | 5.90 |
| sick leave due to depression | 2.00 | 0.63 | 1025 | 3.16 | .002 | 0.76; | 3.24 |
| sick leave due to anxiety disorder | 1.49 | 0.64 | 1025 | 2.34 | .019 | 0.24; | 2.74 |
| sick leave due to somatoform disorder | 0.14 | 0.66 | 1025 | 0.21 | .836 | -1.16; | 1.43 |
| sick leave due to multiple sclerosis | -1.20 | 1.09 | 1025 | -1.10 | .271 | -3.35; | 0.94 |
| time * UC (vs. PNP) (interaction term) | 0.69 | 0.52 | 1025 | 1.33 | .185 | -0.33; | 1.70 |
| time * GP (vs. PNP) (interaction term) | 0.96 | 0.53 | 1025 | 1.82 | .069 | -0.07; | 2.00 |

GP general practitioner program, PNP specialist program (selective care contract in psychiatry, neurology, psychosomatics and psychotherapy, reference group PNP coded with 0), UC usual care, Ste standard error, DF degrees of freedom, CI confidence interval.

**Table 5: Estimated mean differences based on the mixed linear model for the secondary outcome: somatoform symptoms.**

|  | Estimate | Ste | DF | t-value | p-value | 95%-CI | |
| --- | --- | --- | --- | --- | --- | --- | --- |
| intercept | 10.12 | 1.17 | 1026 | 8.62 | <.001 | 7.81; | 12.42 |
| UC vs. PNP | -0.34 | 0.49 | 1026 | -0.69 | .490 | -1.31; | 0.63 |
| GP vs. PNP | -0.57 | 0.56 | 1026 | -1.01 | .314 | -1.67; | 0.54 |
| time [t1] | -1.08 | 0.38 | 1026 | -2.87 | .004 | -1.82; | -0.34 |
| age | 0.05 | 0.02 | 1026 | 3.16 | .002 | 0.02; | 0.09 |
| gender [male] | -1.32 | 0.41 | 1026 | -3.20 | .001 | -2.13; | -0.51 |
| sick leave due to alcohol abuse disorder | -3.48 | 1.78 | 1026 | -1.96 | .050 | -6.96; | 0.01 |
| sick leave due to schizophrenia | -1.24 | 1.71 | 1026 | -0.73 | .468 | -4.60; | 2.12 |
| sick leave due to bipolar disorder | 0.33 | 2.60 | 1026 | 0.13 | .900 | -4.78; | 5.43 |
| sick leave due to depression | 1.55 | 0.74 | 1026 | 2.10 | .036 | 0.10; | 3.01 |
| sick leave due to anxiety disorder | 0.92 | 0.74 | 1026 | 1.24 | .214 | -0.54; | 2.39 |
| sick leave due to somatoform disorder | 1.05 | 0.77 | 1026 | 1.36 | .174 | -0.46; | 2.56 |
| sick leave due to multiple sclerosis | -0.49 | 1.28 | 1026 | -0.38 | .704 | -3.00; | 2.03 |
| time * UC (vs. PNP) (interaction term) | 0.00 | 0.54 | 1026 | -0.01 | .994 | -1.06; | 1.05 |
| time * GP (vs. PNP) (interaction term) | 0.06 | 0.55 | 1026 | 0.10 | .918 | -1.02; | 1.13 |

GP general practitioner program, PNP specialist program (selective care contract in psychiatry, neurology, psychosomatics and psychotherapy, reference group PNP coded with 0), UC usual care, Ste standard error, DF degrees of freedom CI confidence interval.

**Table 6: Estimated mean differences based on the mixed linear model for the secondary outcome: alcohol consumption.**

|  | Estimate | Ste | DF | t-value | p-value | 95%-CI | |
| --- | --- | --- | --- | --- | --- | --- | --- |
| intercept | 2.44 | 0.38 | 1016 | 6.38 | <.001 | 1.69; | 3.20 |
| UC vs. PNP | 0.04 | 0.16 | 1016 | 0.28 | .780 | -0.27; | 0.35 |
| GP vs. PNP | -0.04 | 0.18 | 1016 | -0.24 | .813 | -0.40; | 0.32 |
| time [t1] | -0.21 | 0.11 | 1016 | -2.00 | .046 | -0.42; | 0.00 |
| age | -0.01 | 0.01 | 1016 | -1.65 | .099 | -0.02; | 0.00 |
| gender [male] | 0.94 | 0.13 | 1016 | 7.01 | <.001 | 0.68; | 1.20 |
| sick leave due to alcohol abuse disorder | 2.33 | 0.58 | 1016 | 4.03 | <.001 | 1.20; | 3.47 |
| sick leave due to schizophrenia | -0.51 | 0.56 | 1016 | -0.90 | .369 | -1.62; | 0.60 |
| sick leave due to bipolar disorder | -0.13 | 1.01 | 1016 | -0.13 | .894 | -2.11; | 1.84 |
| sick leave due to depression | 0.11 | 0.25 | 1016 | 0.45 | .650 | -0.37; | 0.60 |
| sick leave due to anxiety disorder | -0.28 | 0.25 | 1016 | -1.14 | .253 | -0.76; | 0.20 |
| sick leave due to somatoform disorder | 0.02 | 0.26 | 1016 | 0.06 | .949 | -0.49; | 0.52 |
| sick leave due to multiple sclerosis | -0.19 | 0.42 | 1016 | -0.45 | .656 | -1.01; | 0.64 |
| time * UC (vs. PNP) (interaction term) | -0.07 | 0.15 | 1016 | -0.48 | .629 | -0.38; | 0.23 |
| time * GP (vs. PNP) (interaction term) | 0.04 | 0.16 | 1016 | 0.25 | .800 | -0.27; | 0.35 |

GP general practitioner program, PNP specialist program (selective care contract in psychiatry, neurology, psychosomatics and psychotherapy, reference group PNP coded with 0), UC usual care, Ste standard error, DF degrees of freedom, CI confidence interval.

**Table 7: Estimated mean differences based on the mixed linear model for patient satisfaction with general practitioners’ care.**

|  | Estimate | Ste | DF | t-value | p-value | 95%-CI |
| --- | --- | --- | --- | --- | --- | --- |
| intercept | 23.17 | 29.85 | 646 | 0.78 | .438 | -35.44; 81.79 |
| UC vs. PNP | -1.67 | 1.87 | 646 | -0.89 | .373 | -5.35; 2.01 |
| GP vs. PNP | -1.70 | 2.28 | 646 | -0.75 | .454 | -6.18; 2.77 |
| age | 0.04 | 0.07 | 646 | 0.60 | .551 | -0.10; 0.18 |
| gender [female] | -6.05 | 1.74 | 646 | -3.47 | .001 | -9.47; -2.63 |
| sick leave due to depression [no] | 6.65 | 3.08 | 646 | 2.16 | .031 | 0.61; 12.70 |
| sick leave due to somatoform disorder [no] | 6.13 | 3.17 | 646 | 1.94 | .053 | -0.08; 12.35 |
| sick leave due to anxiety disorder [no] | 6.37 | 3.16 | 646 | 2.02 | .044 | 0.16; 12.57 |
| sick leave due to alcohol abuse disorder [no] | 2.38 | 6.79 | 646 | 0.35 | .726 | -10.95; 15.72 |
| sick leave due to multiple sclerosis [no] | 2.63 | 5.30 | 646 | 0.50 | .620 | -7.78; 13.04 |
| sick leave due to schizophrenia [no] | 4.57 | 6.63 | 646 | 0.69 | .491 | -8.45; 17.59 |
| sick leave due to bipolar disorder [no] | 18.13 | 20.57 | 646 | 0.88 | .379 | -22.28; 58.53 |
| physical comorbidity | -0.15 | 0.46 | 646 | -0.31 | .755 | -1.06; 0.77 |
| residency [no major cities] | 9.77 | 6.20 | 646 | 1.58 | .115 | -2.40; 21.94 |
| residency [no urban area] | 10.53 | 5.96 | 646 | 1.77 | .078 | -1.17; 22.24 |
| residency [no rural area] | 9.39 | 6.21 | 646 | 1.51 | .131 | -2.80; 21.59 |

GP general practitioner program, PNP specialist program (selective care contract in psychiatry, neurology, psychosomatics and psychotherapy, reference group PNP coded with 0), UC usual care, Ste standard error, DF degrees of freedom, CI confidence interval.

**Table 8: Estimated mean differences based on mixed linear model for patient satisfaction with specialized outpatient care.**

|  | Estimate | Ste | DF | t-value | p-value | 95%- CI |
| --- | --- | --- | --- | --- | --- | --- |
| intercept | 58.44 | 38.48 | 338 | 1.52 | .130 | -17.25; 134.13 |
| UC vs. PNP | -4.36 | 2.73 | 338 | -1.60 | .111 | -9.72; 1.01 |
| GP vs. PNP | -1.06 | 3.58 | 338 | -0.30 | .797 | -8.11; 5.98 |
| age | 0.38 | 0.11 | 338 | 3.40 | .001 | 0.16; 0.60 |
| gender [female] | 0.15 | 2.65 | 338 | 0.06 | .955 | -5.05; 5.36 |
| sick leave due to depression [no] | -4.79 | 4.19 | 338 | -1.14 | .254 | -13.03; 3.45 |
| sick leave due to somatoform disorder [no] | -0.24 | 4.41 | 338 | -0.05 | .957 | -8.91; 8.43 |
| sick leave due to anxiety disorder [no] | -1.60 | 4.29 | 338 | -0.37 | .710 | -10.02; 6.83 |
| sick leave due to alcohol abuse disorder [no] | -7.20 | 10.28 | 338 | -0.70 | .484 | -27.41; 13.02 |
| sick leave due to multiple sclerosis [no] | -12.48 | 6.26 | 338 | -1.99 | .047 | -24.80; -0.17 |
| sick leave due to schizophrenia [no] | -5.64 | 7.00 | 338 | -0.81 | .421 | -19.41; 8.13 |
| sick leave due to bipolar disorder [no] | 6.63 | 22.40 | 338 | 0.30 | .767 | -37.43; 50.69 |
| physical comorbidity | 0.36 | 0.69 | 338 | 0.52 | .603 | -1.00; 1.72 |
| residency [no major cities] | 11.50 | 10.36 | 338 | 1.11 | .268 | -8.87; 31.87 |
| residency [no urban area] | 8.14 | 10.01 | 338 | 0.81 | .417 | -11.56; 27.83 |
| residency [no rural area] | 10.70 | 10.36 | 338 | 1.03 | .302 | -9.68; 31.09 |

GP general practitioner program, PNP specialist program (selective care contract in psychiatry, neurology, psychosomatics and psychotherapy, reference group PNP coded with 0), UC usual care, Ste standard error, DF degrees of freedom, CI confidence interval.

**Table 9: Estimated mean differences based on the mixed linear model for patient satisfaction with outpatient psychotherapy.**

|  | Estimate | Ste | DF | t-value | p-value | 95%-CI |
| --- | --- | --- | --- | --- | --- | --- |
| intercept | 62.57 | 34.09 | 271 | 1.84 | .068 | -4.55; 129.68 |
| UC vs. PNP | 1.38 | 2.84 | 271 | 0.49 | .627 | -4.20; 6.96 |
| GP vs. PNP | -2.31 | 3.59 | 271 | -0.64 | .521 | -9.37; 4.76 |
| age | -0.01 | 0.11 | 271 | -0.13 | .895 | -0.23; 0.20 |
| gender [female] | 1.50 | 2.73 | 271 | 0.55 | .582 | -3.86; 6.87 |
| sick leave due to depression [no] | -2.08 | 4.38 | 271 | -0.48 | .635 | -10.71; 6.55 |
| sick leave due to somatoform disorder [no] | -0.89 | 4.62 | 271 | -0.19 | .847 | -9.98; 8.20 |
| sick leave due to anxiety disorder [no] | 3.26 | 4.14 | 271 | 0.79 | .431 | -4.89; 11.42 |
| sick leave due to alcohol abuse disorder [no] | -0.89 | 10.14 | 271 | -0.09 | .930 | -20.85; 19.07 |
| sick leave due to multiple sclerosis [no] | -13.86 | 12.77 | 271 | -1.09 | .279 | -38.99; 11.27 |
| sick leave due to schizophrenia [no] | 1.70 | 9.29 | 271 | 0.18 | .855 | -16.58; 19.98 |
| sick leave due to bipolar disorder [no]* | - | - | 271 | - | - | - |
| physical comorbidity | 0.17 | 0.70 | 271 | 0.24 | .810 | -1.22; 1.55 |
| residency [no major cities] | 16.27 | 12.31 | 271 | 1.32 | .187 | -7.95; 40.50 |
| residency [no urban area] | 13.34 | 11.98 | 271 | 1.11 | .267 | -10.25; 36.92 |
| residency [no rural area] | 12.84 | 12.25 | 271 | 1.05 | .295 | -11.27; 36.96 |

GP general practitioner program, PNP specialist program (selective care contract in psychiatry, neurology, psychosomatics and psychotherapy, reference group PNP coded with 0), UC usual care, Ste standard error, DF degrees of freedom, CI confidence interval.

**Note:* There was no variance in the diagnosis of bipolar disorder in this subsample.
